# Meiosis in Quarantine discussions lead to an action plan to increase diversity and inclusion within the genetics community

**DOI:** 10.1371/journal.pgen.1009648

**Published:** 2021-07-15

**Authors:** Katherine K. Billmyre, María Angélica Bravo Núñez, Douglas K. Bishop, Francesca Cole

**Affiliations:** 1 Stowers Institute for Medical Research, Kansas City, Missouri, United States of America; 2 Department of Molecular and Cellular Biology, Harvard University, Cambridge, Massachusetts, United States of America; 3 Department of Radiation and Cellular Oncology; Department of Molecular Genetics and Cell Biology, University of Chicago, Chicago, Illinois, United States of America; 4 Department of Epigenetics and Molecular Carcinogenesis, The University of Texas MD Anderson Cancer Center, Smithville, Texas, United States of America; The University of North Carolina at Chapel Hill, UNITED STATES

Due to the ongoing COVID-19 pandemic, virtually-held conferences have emerged as a safe substitute for community-wide scientific discussion. The meiosis community held the virtual webinar series, “Meiosis in Quarantine” that included a forum to discuss diversity and equity issues with the goal of providing actionable items to foster the inclusion of underrepresented groups in annual meetings, labs, institutions, and the scientific community.

In this Opinion Piece, we take a broad definition of the term ‘underrepresented groups’ to promote inclusion. Our definition includes, but is not limited to, individuals from underrepresented gender, racial, and/or ethnic groups, but also neurodiverse individuals, those with disabilities, members of the LGBTQ+ community, those from disadvantaged backgrounds, and first-generation students for whom neither parent completed a bachelor’s degree. For a more inclusive definition refer to https://grants.nih.gov/grants/guide/notice-files/NOT-OD-20-031.html.

The discussion consisted of two sessions. In the first session, trainees (technicians, graduate students, and postdocs) discussed the barriers encountered by underrepresented individuals in science and highlighted active measures to address these barriers. Specifically, this discussion focused on the shortage of diversity in academia [1], the poor retention and visibility of underrepresented groups in academia [2–5], and the financial burden these groups encounter [2,5–8]. In the second session, trainees’ concerns and solutions were discussed with PIs and group leaders to develop a strategic diversity and inclusion action plan for the meiosis community, and more broadly for the communities we are part of (*e*.*g*., genetics). Importantly, for any intervention to be effective it requires a holistic approach—within the community and our research institutions. This perspective piece summarizes many of the opinions expressed at the two ‘Meiosis in Quarantine’ discussions.

## Part 1: Increasing diversity and inclusivity at conferences

**Fig 1:**
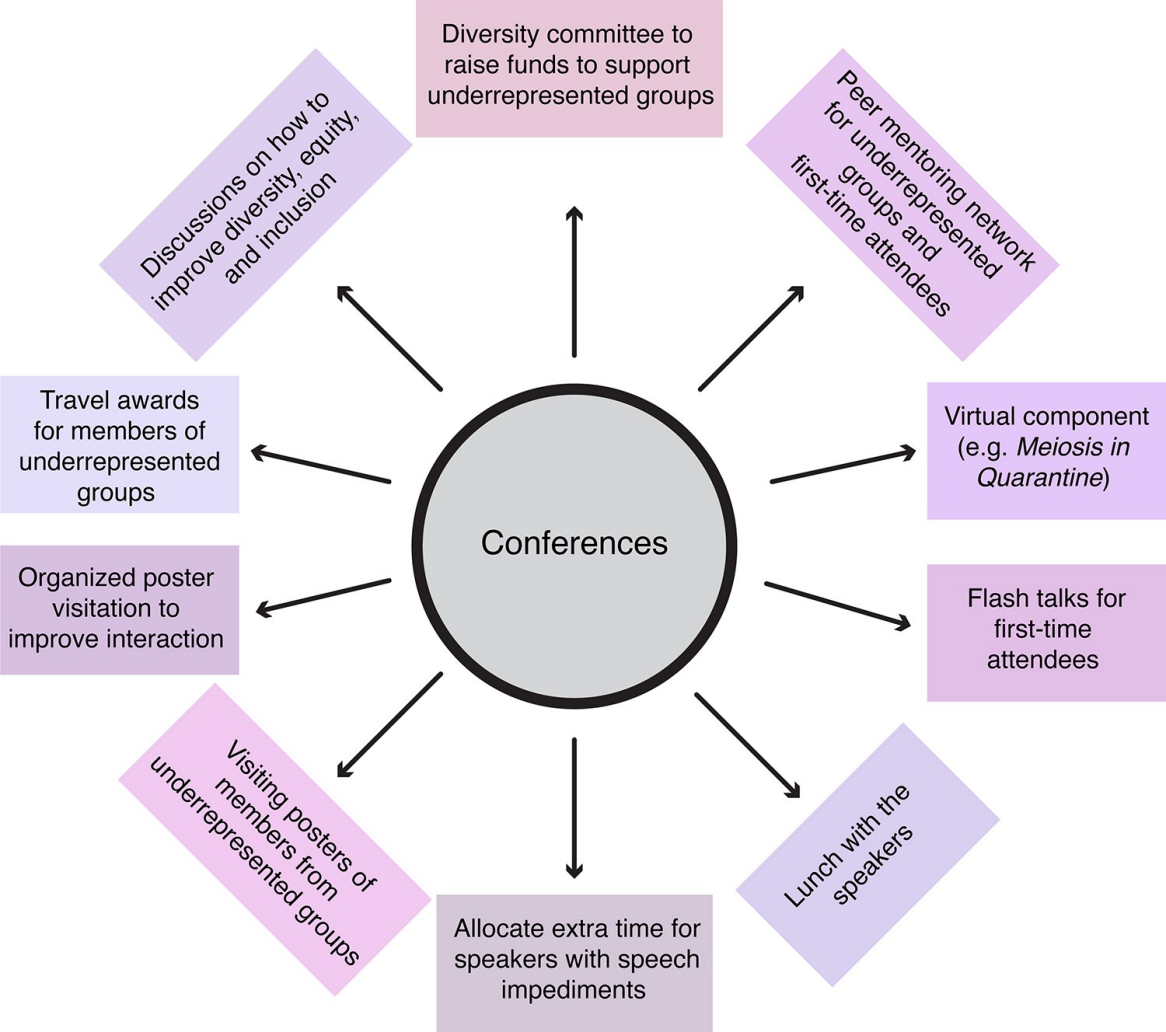
Concrete examples to promote diversity and inclusion at conferences. These are action items for future conferences that were discussed during the ‘Meiosis in Quarantine’ equity and inclusion sessions.

Given that innovation in research is fueled by the diversity of its scientists [9–11] and that we consider fair treatment of all members of our community to be a moral duty, approaches need to be developed to welcome and support diversity in our community. Unfortunately, despite many efforts, several conferences struggle to foster diversity and inclusivity in both attendance and invited speakers [12–15]. This is particularly harmful to underrepresented scientists as failure to include underrepresented groups in invited speaker lists, or amongst plenary speakers, can further compound the lack of diversity by alienating the remaining diverse trainees [12,16]. The main goals of the discussions were to identify active measures to increase representation of underrepresented groups and to create an inclusive environment at conferences. Summarized below are examples of actionable items we propose the genetics communities take to improve equity at conferences (**Fig 1**).

### Diversifying speaker lists

Conferences frequently lack diverse presenters [12–15]. We recommend that organizers examine their list of invited speakers with the express purpose of ensuring adequate representation and to determine if it is representative of the general population. To increase adequate representation, we suggest that organizers consider inviting diverse postdocs and graduate students, given the scarce representation of some groups at the faculty level. We also encourage group leaders/PIs to be allies by inquiring about the diversity of speaker lists before agreeing to speak. By being active allies, we can increase representation of underrepresented groups presenting at our conferences. For more strategies to increase speaker diversity see [17].

### Barriers to in-person attendance

In addition to increasing diversity in conference programming, we can also make conferences available and accessible to members of all groups. Some barriers are limited financial resources [18], teaching/administrative burdens [19], and dangers traveling as a member from an underrepresented group (*e*.*g*., https://www.humandignitytrust.org/lgbt-the-law/map-of-criminalisation). For example, feedback from online conferences suggests that having a digital component (*e*.*g*., recordings) can increase accessibility by reaching audiences that would be unable to attend otherwise, those who have disabilities, and those who are neurodiverse [20]. Additionally, conferences can support the attendance of underrepresented individuals by raising funds for those individuals and by encouraging invited speakers who do not need the funding to return their travel money on the condition it supports the travel of diverse trainees (**Fig 1)**.

### Inequities in networking opportunities

Our discussions revealed that trainees feel that inequalities in resources, publishing, and networking are barriers to retention of young scientists. We suggest that providing equal opportunities to diverse trainees to present at conferences, write reviews/research articles, and engage in networking events may potentially help retain trainees in our community. An example of this inequality is that trainees who do not descend from ‘science power houses’ may experience lower attendance at their posters/talks. As a result, membership in prestigious and well-connected lab lineages amplifies disparities in the community. To promote interactions within the community, we encourage organizers of conferences to design structured networking schemes that encourage all scientists to interact either at random or in a categorized fashion, thereby encouraging the inclusion of underrepresented individuals (**Fig 1**). Examples of innovative networking schemes include the “meet the speakers/experts” lunch at FASEB conferences and the name tag ribbons at Genetics Society of America conferences that allow attendees to proudly declare groups they identify with and their goals (*e.g.*, on the job market, recruiting trainees, editor) encouraging interactions.

Further action items are summarized in (**Fig 1**). Together, these action items are some of the critical steps necessary to achieve equity at conferences and to create an environment wherein individuals from underrepresented groups feel welcome in our communities.

## Part 2: Diversity and equity beyond conferences

**Fig 2:**
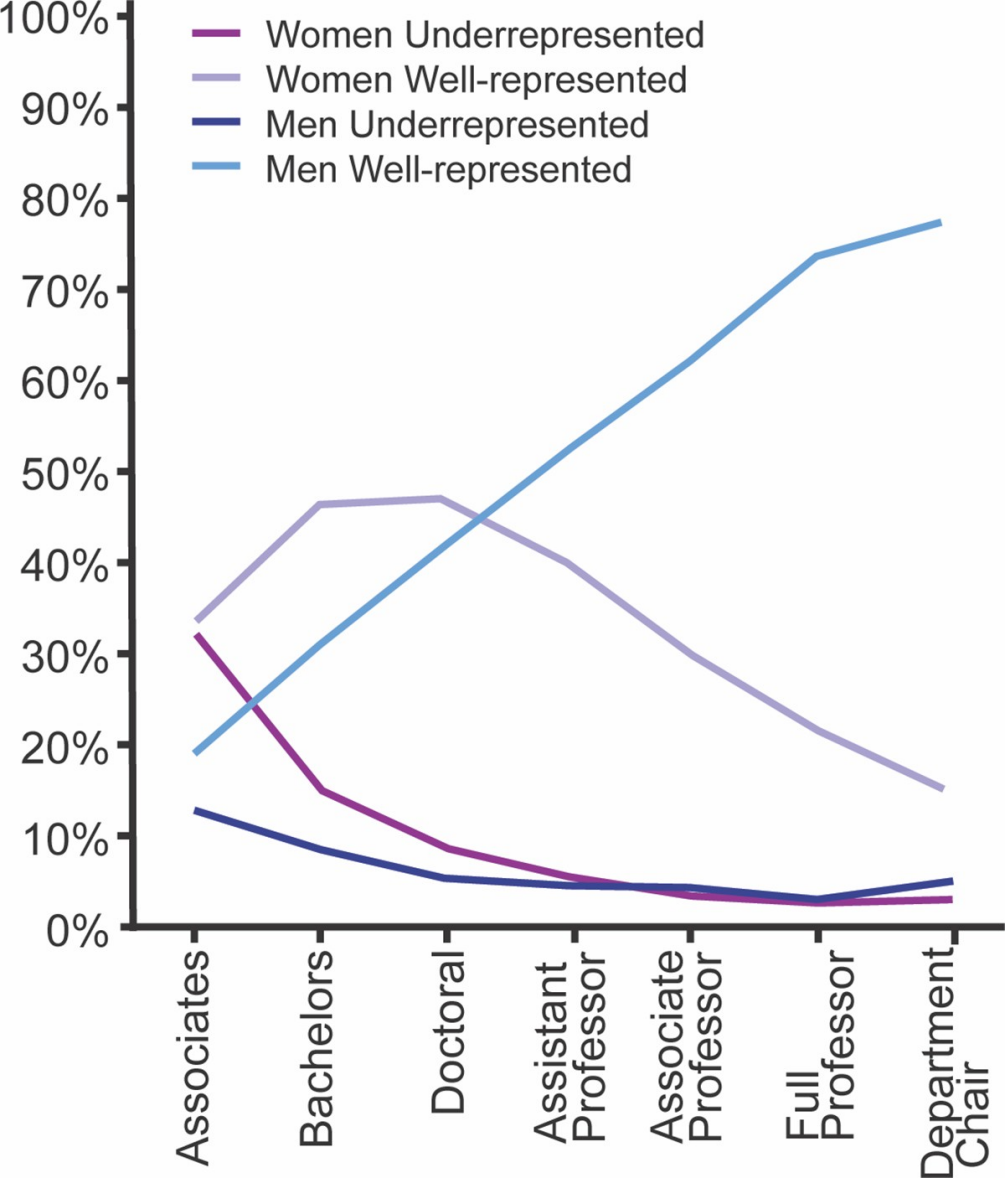
Percent of underrepresented groups compared to well represented groups at different career stages. Underrepresented individuals (as defined in [51]) are lost at multiple academic career stages. Data were compiled from the Fall 2017 and Fall 2018 US Department of Education, National Center for Education Statistics, Integrated Postsecondary Education Data System (IPEDS), and the Association of American Medical Colleges Faculty Roster, 2018. Figure adapted from [51].

Initiatives to support young scientists from underrepresented groups have increased the proportion of diverse scientists in biomedical research in the past three decades [21,22]. However, the current PI pool, which is primarily white and male at biomedical institutions, is not reflective of the student population [22,23] (**Fig 2**). There remains extremely low visible representation of other groups including people of color, members of the LGBTQ+ community, people with physical disabilities, and neurodiverse individuals [24–27]. The lack of representation of underrepresented individuals in leadership positions (*e*.*g*., department chairs, PIs, and group leaders) can discourage diverse trainees from pursuing academic careers [28–30]. For example, Zambrana et al provide strategies to improve mentoring and retention of underrepresented faculty [28]. In the rest of this piece, we discuss barriers and action items that were identified during the ‘Meiosis in Quarantine’ sessions to increase diversity and equity in our own labs and home institutions.

### Immediate actions to improve inclusivity

The following sections will focus on strategies that can be implemented at the level of individuals, labs, and departments/programs with relatively little financial cost.

### Early STEM outreach

Increasing interest in STEM at the K-12 and undergraduate level through education outreach can increase the number of underrepresented individuals interested in pursuing a STEM career [31,32]. We recommend that individual mentors and programs support K-12 education outreach opportunities for trainees and faculty, as it is beneficial to the community and good teaching/mentoring experience for trainees. In addition, we recommend that undergraduate programs have freshman informational sessions highlighting graduate school experiences and research opportunities. Lastly, recruitment efforts at meetings such as Society for Advancement of Chicanos/Hispanics and Native Americans in Science (SACNAS) and Annual Biomedical Research Conference for Minority Students (ABRCMS) can increase student interest in graduate programs and provide support for those applications [33]. During the discussion session, the meiosis community proposed having a booth at these meetings to generate interest in the field of genetics and to increase community diversity.

### Removing barriers to graduate school

**Fig 3:**
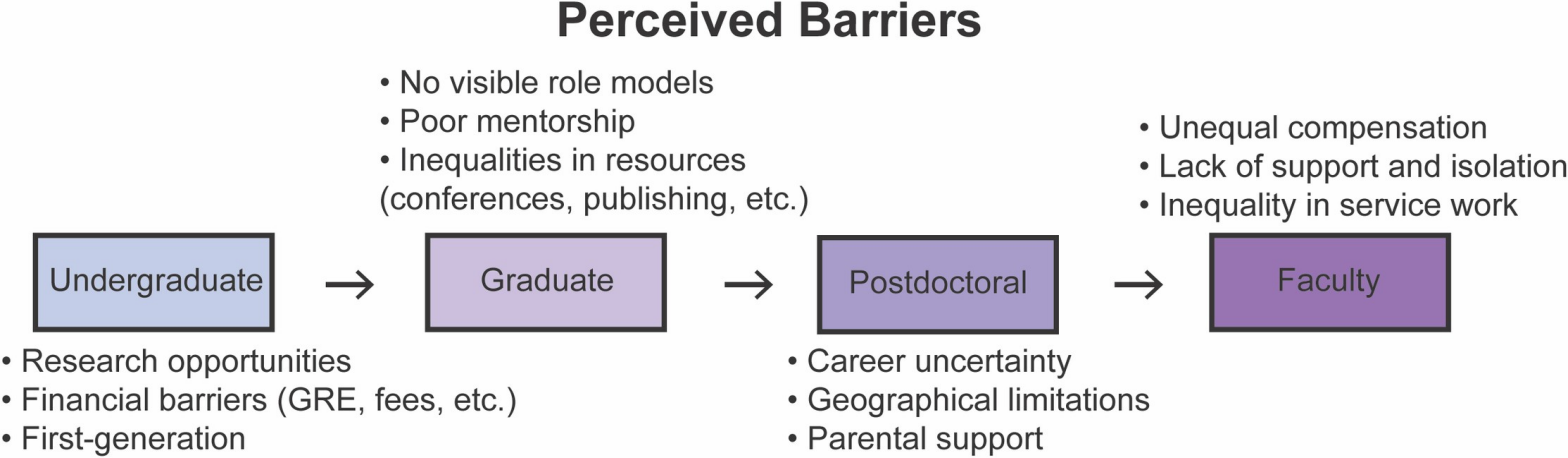
Barriers that were raised by trainees during the ‘Meiosis in Quarantine’ discussion broken down by career stage.

The trainees discussed barriers that they felt discouraged or impeded underrepresented groups from entering graduate school. The first of these discussions centered around undergraduates gaining research experience that is a critical gateway to graduate school (**Fig 3**). We encourage lab leaders to set up undergraduate positions in a way to create opportunities to participate in research versus only lab grunt work (*e*.*g*., making plates, cleaning dishes) and provide authorship credit when they have contributed data and/or analysis. Underrepresented groups may not be aware that they deserve credit for their contributions and may not advocate for themselves [34].

Financial barriers to graduate school can be eliminated. Research experience is usually unpaid or underpaid, thereby excluding economically disadvantaged students who cannot afford to work for little or no salary. Because of this, we recommend normalizing compensation for undergraduates with economic needs either by increasing work study opportunities to include research or paying minimum wage. Increased compensation will increase diversity in STEM through expanding the number of research opportunities for those with financial barriers [7,8]. In addition, the financial cost of the Graduate Record Examination (GRE) and application fees are barriers during the graduate school admissions process (**Fig 3**) [33,35]. Although the GRE requirement is becoming less frequent, we recommend that graduate programs that have not yet done so, abolish the GRE requirement as it discriminates against underrepresented groups and is not predictive of career success [36,37]. Given that many applicants are not aware of the option of waiving application fees or are concerned about negative consequences associated with asking for a waiver, we recommend graduate programs have clear policies for need-based fee waivers or eliminate fees entirely and that they make such policies transparent. Additionally, graduate programs need to be transparent about stipend and relocation costs as underrepresented individuals may be disproportionately less able to afford relocating [38].

Importantly at every stage of career development, the community needs to make academia more accessible for students from non-academic backgrounds who are not familiar with the academic system [39]. This lack of knowledge can put students at a disadvantage and prevent underrepresented students from joining the scientific community. Small efforts can be beneficial, such as providing guidance on application materials and informing students early via outreach that most biomedical PhD programs do not require payment of tuition and provide a livable stipend. For more strategies to recruit and retain diverse students see [33].

### Improving mentorship

During the discussion, trainees reported that they felt having excellent mentorship was critical for success [40]. For trainees, we recommend establishing a broad mentorship network (peer mentors, committee members, and other faculty) as not every advisor will provide effective mentorship in all areas (see for more resources: https://www.nap.edu/resource/25568/interactive/). To increase the quality of faculty mentorship, we encourage departments/institutions to provide formal training to lab leaders in mentoring, including strategies to improve mentoring of diverse students [41,42]. This will help mentors identify and reduce potential problems that diverse trainees may face. Resources for mentoring networks/programs of underrepresented groups are provided in [43] and at the following organizations National Research Mentoring Network (NRMNet) (https://nrmnet.net/nrmn-resources/), Center for Improvement of Mentored Experiences in Research (CIMER) (https://cimerproject.org/training/), and Future of Biomedical Graduate and Postdoctoral Training 2 (FOBGAPT2) (https://gs.ucdenver.edu/fobgapt2/main.php).

Further, we recommend that graduate programs adopt a formal peer mentoring system to facilitate interactions with peers, as well as assign neutral faculty advisors to provide additional guidance. Graduate programs should establish mentoring quality standards and enforce those standards by revoking the PI’s privilege of accepting students in their laboratory. Additionally, we strongly encourage tenure committees to consider mentorship and outreach as part of faculty evaluations for hiring, promotion, and tenure. Just as teaching and service to the community have been suggested to be important parts of the tenure package, mentorship and outreach should be too [44].

### Improving laboratory culture

It was discussed in the trainee session that in some labs and graduate programs there is a toxic culture surrounding excessive work hours and unrealistic expectations of productivity. PIs should establish a formal mentoring contract that clearly delineates expectations for both the mentor and mentee and these contracts should be re-evaluated regularly. Environments that establish unrealistic goals (whether perceived or real) can lead to mental health issues for trainees [45]. This can be particularly damaging for trainees who are often dependent on their advisors for success and are unable to leave unhealthy environments. These types of environments may have a disproportionate impact on underrepresented individuals and advisors should be aware that trainees may benefit from different mentoring styles and support [46]. To improve mental health and work environments in STEM, trainees need good role models of healthy work-life balance both from their faculty and their peers.

### Supporting parents

The lack of support and the active macro- and microaggressions against people with children has led graduate students and postdocs with familial obligations to be forced out of academia [47]. This issue is more prevalent as the training period becomes longer, and the age at which women obtain faculty jobs is past the optimal childbearing age. For example, Morgan et al quantify how scholarship is affected by parenthood and identify strategies to increase retention [48]. We recommend that advisors and departments work to create a family friendly culture, for example, by including families at work social events. Community support for parents can include actions such as, scheduling meetings during daycare hours, support for shipping breastmilk home from conferences, pumping rooms, and flexible workhours [48–50].

### Faculty hiring

Importantly, the largest drop in most underrepresented groups in academia is in the transition from postdoctoral researcher to a tenure-track position, with women being disproportionately affected [2,3,51]. Women are more likely to be in a dual-career relationship with an academic [52] and have increased childcare responsibilities making it harder to navigate the academic hiring process. Schiebinger et al is a comprehensive resource of partner patterns in academia providing valuable information regarding dual-career couples [52]. This hurdle is likely to be higher for women of color who may be more likely to be impacted by cultural barriers and limitations on their geographic location [53]. Increasing support for underrepresented junior faculty during and after the hiring stage through solutions discussed below will help increase retention of scientists after the postdoctoral level.

One of the issues underrepresented groups face is inequality during the hiring and negotiation process [54]. Some institutions have variable salaries but even at those with clear salary tiers, startup package negotiations can lead to unequal resources (*e*.*g*., funds, lab space, etc.) for new faculty and unseen inequality between faculty. This inequality can create large disparities in productivity and affect retention and tenure [55]. To help ameliorate this, we recommend that negotiation training for graduate students and postdocs become a standard part of their training. We also strongly encourage senior faculty to advocate for junior colleagues to prevent disparities. Furthermore, departments and institutions should develop consistent, fair, and transparent hiring processes.

## Longer term actions to combat systemic exclusion

Unfortunately, many of the barriers that were discussed during the sessions require extensive institutional reform. This type of reform is expensive, time consuming, and requires buy in at the institutional level, which can be hard to establish. The following sections outline some of the action items requiring reform at the institutional level.

### Benefits for caregivers

Institutions need to have both maternity and paternity leave for graduate students and postdocs as current polices are often not clear or very minimal [56]. This is especially an issue at the postdoctoral level as postdocs are usually on yearly contracts and have fewer benefits compared to permanent employees. Herschberg et al highlight the problems of haphazard institutional policies for postdocs [57]. Furthermore, there are examples of active discrimination against women of childbearing age as some advisors are worried about the loss of productivity that occurs with family leave and whether trainees will be ‘serious’ about their careers [47]. One action item that was discussed as a solution to increase retention of trainees who feel they must choose between academia and children is to provide more financial support (either internally or externally) to support a technician to cover a trainee’s work during family leave. For example, the NIAID has a program to give a supplement for this purpose (https://grants.nih.gov/grants/guide/pa-files/PA-18-926.html), we encourage other grant giving institutions to support similar initiatives. More recently, NIH issued a notice providing graduate students and postdocs that hold a Ruth L. Kirschstein NRSA access to funds to support childcare costs (https://grants.nih.gov/grants/guide/notice-files/NOT-OD-21-070.html). Increasing support during family leave will also help combat hiring discrimination that occurs against trainees who are perceived to want children.

Importantly, the community needs to be cognizant that caregiving is not only for parents. Many individuals have family responsibilities to care for elders or extended family. This is made more challenging by the scientific culture of favoring candidates with repeated long-distance moves during competitions for grants and jobs [58]. This culture is likely to be damaging and potentially impact underrepresented trainees disproportionately. Changing this culture will help retention of underrepresented trainees and parents while making long-term academic careers more sustainable for everyone.

### Retention at the faculty level

One of the main barriers to the development of diversity in academic communities is a lack of retention of diverse members due to structural and social/psychological barriers [59]. For example, the scarcity of underrepresented individuals in the scientific community may deter feelings of inclusion [9,60]. Hence, increasing the number of diverse individuals in our labs and institutions is absolutely critical to achieving retention of these groups in academia. To specifically increase retention of diverse faculty in tenure-track positions, we recommend deliberate and targeted recruitment of postdocs from underrepresented groups with dedicated financial and space commitments from institutional leadership. One way to accomplish this is through cluster hires as they can promote diversity and support inclusion and retention. Flaherty describes the substantial benefits to the establishment and maintenance of diversity using a cluster hire [61]. Other strategies to enhance diversity in faculty positions have been highlighted and discussed in [6]. Finally, these efforts should not be viewed as fulfilling a quota or an imposition of a new source of bias, but instead as a measure to counterbalance long-standing biases that have hampered the inclusion and promotion of brilliant colleagues.

### Inclusivity of ALL underrepresented groups

Underrepresentation of women and people of color are often a point of discussion at diversity and inclusion events. However, there are many groups left out of the discussion. These groups include, but are not limited to, members of the LGBTQ+ community, people with disabilities, first generation students, and neurodiverse individuals. People with disabilities can find themselves excluded from labs that are not physically set up to accommodate them [25]. Furthermore, those with disabilities can be at a disadvantage at events such as large conferences that require standing and walking for long periods of time. As a community, we need to be aware of seen and unseen disabilities and work to increase access to labs, conferences, and institutions. It should also be noted that members of other underrepresented groups are not easily identified and may be afraid to ‘out’ themselves for fear of discrimination. As a result, the issues these groups face are mostly unknown and unrecognized. A great example of change occurring within the publishing community is the creation of new policies to allow name changes on publications for transgender and non-binary individuals [62,63]. Policies like this help educate the community about barriers particular groups face while providing a solution. However, there are many bigger steps which need to be taken to make members of these often-overlooked groups feel welcome in science.

As a community we need to be more welcoming to those who are different from ourselves and open a dialogue to become allies for those who are discriminated against. The more we have discussions in truly inclusive and safe environments, and create and follow through on action plans, the more likely substantial change will occur in our community. As the genetics community contains members from many subfields, we have the opportunity to take a leadership role in diversifying the scientific community across disciplines in a way that will benefit all of us and increase the quality and innovation occurring throughout STEM.
